# Birt–Hogg–Dube syndrome: A case report and case study of primary spontaneous pneumothorax caused by folliculin gene mutation

**DOI:** 10.1097/MD.0000000000046188

**Published:** 2025-11-21

**Authors:** Xu Shen, Hu Liao

**Affiliations:** a Department of Thoracic Surgery, Chengdu-Shangjin Nanfu Hospital, Chengdu, China; b Department of Thoracic Surgery, West China Hospital, Sichuan University, Chengdu, China.

**Keywords:** Birt–Hogg–Dube syndrome, case report, folliculin gene mutation, pulmonary cystic lesions, recurrent pneumothorax

## Abstract

**Rationale:**

Birt–Hogg–Dube (BHD) syndrome is a rare autosomal dominant disorder characterized by a triad of manifestations, including recurrent spontaneous pneumothorax, multiple cutaneous fibrofolliculomas, and renal neoplasms. Familial clustering of spontaneous pneumothorax should raise clinical suspicion of BHD syndrome. This syndrome is caused by pathogenic variants of the folliculin gene (FLCN gene) encoding folliculin, which predisposes to pneumothorax through dysregulated matrix metalloproteinase activity, leading to elastic fiber degradation in the pulmonary parenchyma.

**Patient concerns:**

A 28-year-old female presented with recurrent pneumothorax and a family history of primary spontaneous pneumothorax. Emergency video-assisted thoracoscopic surgery with bullectomy and cyst resection was performed.

**Diagnoses:**

The FLCN gene mutation was confirmed by genetic testing after surgery. The clinicopathological characteristics, molecular genetics, and therapeutic strategies for BHD syndrome have been discussed in the literature. The female who experienced 3 episodes of spontaneous pneumothorax within 24 months. Notably, her multigenerational family history included her paternal uncle and cousins affected by primary spontaneous pneumothorax.

**Interventions:**

Emergency video-assisted thoracoscopic surgery revealed multiple subpleural cysts (3–15 mm in diameter), predominantly in the lower lung lobes, requiring bullectomy and cyst wall resection. Histopathological analysis revealed characteristic thin-walled cysts lined with alveolar epithelium. Postoperative genetic sequencing revealed a heterozygous splice-site variant in FLCN gene, establishing a diagnosis of BHD syndrome.

**Outcomes:**

After regular follow-up, the patient had no recurrence of spontaneous pneumothorax, such as bullae rupture, until recently. This case highlights the importance of integrating radiological findings with molecular diagnostics for the evaluation of recurrent pneumothorax. It is particularly necessary to emphasize that the patient and her legal guardian has given us informed consent. Chengdu-Shangjin Nanfu Hospital ethics Committee has approved this study.

**Lessons:**

This case shows a typical phenomenon of family clustering that should raise clinical suspicion of BHD syndrome. BHD syndrome is a rare autosomal dominant genetic disorder that caused by mutations in FLCN gene. The gene is a tumor suppressor gene that encodes the follicle-stimulating hormone folliculin. Follicle-stimulating hormone folliculin is expressed in most tissues, including the skin and its appendages, type I alveolar epithelial cells, and distal renal tubular epithelial cells, which explains why BHD syndrome occurs in the skin, lungs, and kidneys. The literature review shows that there are 12 similar cases, including the male-to-female ratio and average age. Thoracoscopic surgery had the best effect, followed by conservative treatment, and the recurrence rate was higher after closed thoracic drainage. Due to the rarity of this syndrome, this article reminds clinicians to pay attention to its familial clustering characteristics during diagnosis and treatment, and also reminds other family members to pay attention to screening for the clinical features related to this syndrome, so as to facilitate early treatment. Furthermore, this case also provides significant reference value for identifying the causes and molecular mechanisms of spontaneous pneumothorax, especially those with a familial clustering tendency.

## 1. Introduction

Birt–Hogg–Dube (BHD) syndrome is a rare genetic disorder that was 1st identified by Birt in 1997. Pneumothorax and lung cysts are the main manifestations of lung disease.^[1]^ Studies have shown that patients with BHD syndrome are 32 times more likely to develop spontaneous pneumothorax.^[2]^ Other clinical manifestations of BHD syndrome include multiple cutaneous fibrocystadenomas and kidney tumors etc. Colorectal adenoma, colorectal cancer, parotid eosinoma, parathyroid eosinoma, etc have also been reported in patients with BHD syndrome. However, the causal relationship between BHD syndrome and these diseases remains unclear, and more studies are needed to confirm these associations.^[3]^

BHD syndrome is caused by mutations in folliculin gene (FLCN gene). The FLCN gene, located in 17p11.2, is a tumor suppressor gene that encodes the follicle-stimulating hormone folliculin.^[4]^ The FLCN gene comprises 14 exons, of which exons 4 to 14 are translatable, and approximately 44% of these mutations are frameshift mutations caused by insertion or deletion of cytosine in exon 11, which is considered to be the FLCN gene mutation hotspot.^[5,6]^ Follicle-stimulating hormone folliculin, a protein encoded by the FLCN gene, is expressed in most tissues, including the skin and its appendages, type I alveolar epithelial cells, and distal renal tubular epithelial cells, which explains why BHD syndrome occurs in the skin, lungs, and kidneys. Studies have confirmed that when the FLCN gene is mutated, macrophages and fibroblasts secrete a large number of inflammatory factors, which eventually cause the destruction of lung elastic fibers, and lead to pneumothorax.^[4]^

## 2. Case presentation

**Figure 1.**
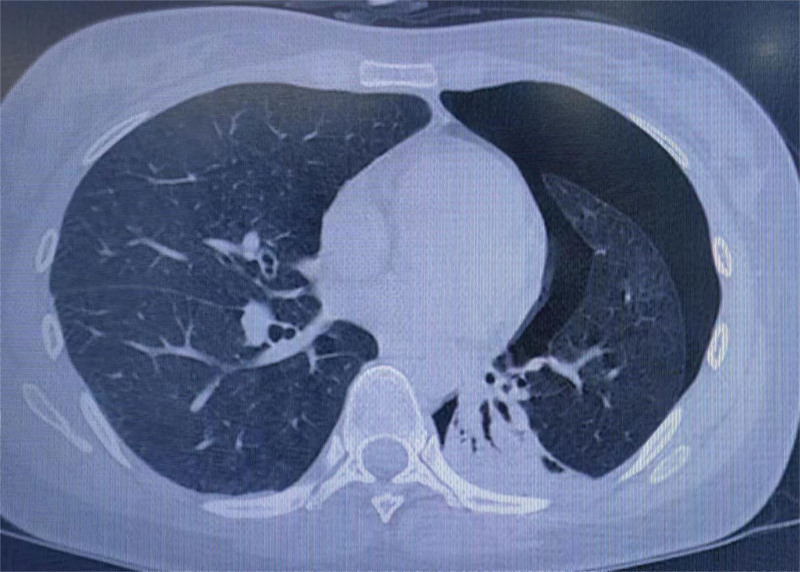
Preoperative computed tomography findings.

**Figure 2.**
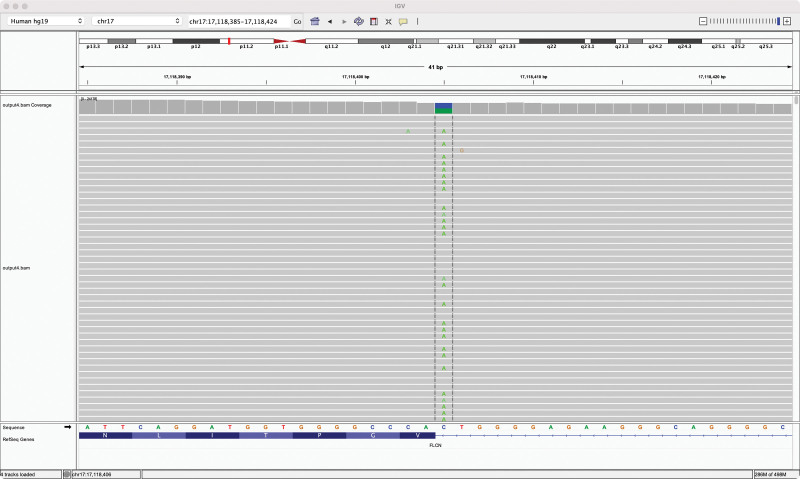
Folliculin gene locus map.

**Figure 3.**
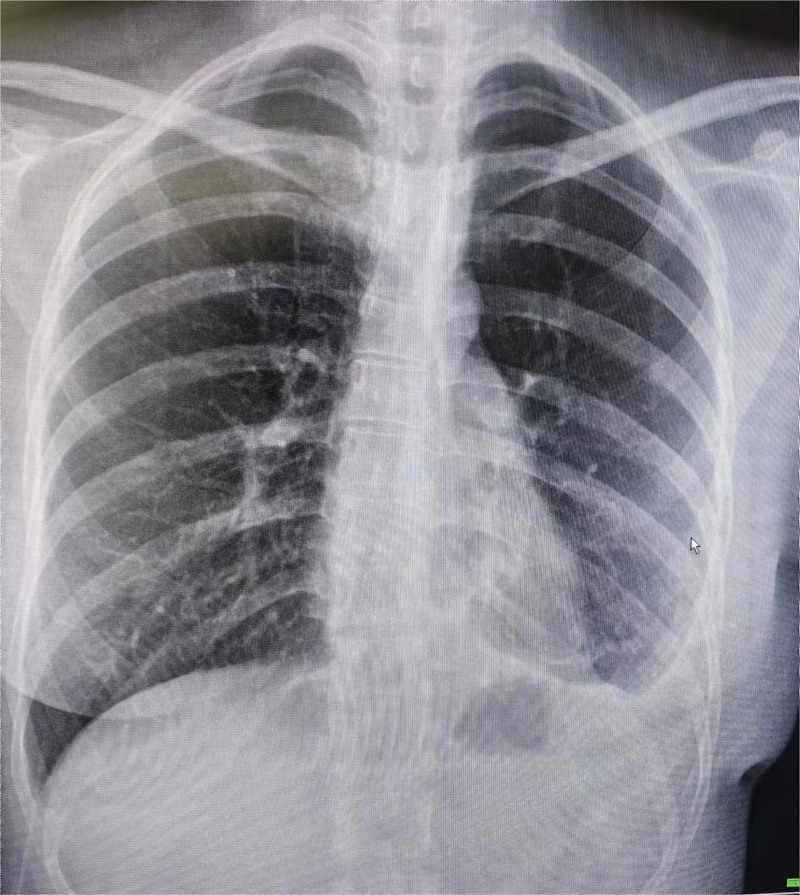
Postoperative chest digital radiography positive performance.

The 28-year-old female patient was admitted to the hospital due to repeated left chest and back pain discomfort for 7 days, aggravated with shortness of breath for 2 days. Then she came to our hospital’s emergency department. Chest computed tomography (CT) in the emergency department showed left hydropneumothorax, mainly pneumatosis, compressed by more than 60% (Fig. 1). Physical examination: visual inspection showed that the left thorax was slightly full, palpation on the left side of the tactile language tremor weakened, percussion on the affected side of the drum sound, auscultation, the breathing sound on the left side weakened, and no obvious abnormalities were found in other physical examinations. Past medical history: the patient with left pneumothorax underwent left thoracic closed drainage 3 years prior and was discharged from the hospital after recovery and extubation, which was regarded as the second episode. Family history: his father had a history of bilateral bullae; both her cousin and uncle (her cousin’s father) had bilateral spontaneous pneumothorax, and they were diagnosed with BHD syndrome, a rare autosomal dominant genetic disease in foreign hospitals and abroad. The patient had a recurrence of spontaneous pneumothorax with obvious symptoms, and CT indicated 60% compression of the left lung. The patient had obvious family genetic tendencies, and BHD syndrome was diagnosed in the family members. The surgical indications were obvious. Then we admitted the patient to the hospital and carried out the necessary preoperative preparations. Thoracoscopic left lower lobe lung bulla resection, left lower lobe lung cyst resection, and pleural fixation were performed under general anesthesia. Intraoperative findings: there was a small amount of adhesion and effusion in the patient’s left thoracic cavity. A lung cyst of about 1.5 × 1 cm in size was seen in the basal segment of the left lower lobe, and a lung bulla was seen in the proximal oblique fissure of the left lower lobe. Postoperative recovery was smooth, and the patient was discharged from the hospital on the 4th day. The patient’s blood samples were collected for genetic testing. A rare variant FLCN gene alternative splicing mutation was detected, and the mutation position was located in the sequence of exon 13. BHD syndrome was also confirmed (see Fig. 2). After regular follow-up, the patient had no recurrence of spontaneous pneumothorax, such as bullae rupture, until recently (Fig. 3).

## 3. Discussion

The familial distribution of primary spontaneous pneumothorax was 1st reported in 1921,^[7]^ and its mode of inheritance has since been debated. Currently, known genetic disorders associated with primary spontaneous pneumothorax include BHD syndrome, alpha-antitrypsin deficiency, Marfan syndrome, homocystinuria, and Ehlers–Danlos syndrome. Many experiments have shown that, among these, the FLCN gene in BHD syndrome is the most closely related gene in familial spontaneous pneumothorax. Pneumothorax associated with genetic diseases such as alpha-antitrypsin deficiency, Marfan syndrome, homocystinuria, and Ehlers–Danlos syndrome is quite rare. In BHD syndrome, familial primary spontaneous pneumothorax was observed,^[8]^ and in a large sample family survey of BHD syndrome by Schmidt et al, 32% of patients had a history of spontaneous pneumothorax, and 85% had bullosa found on CT.^[9]^ Painter reported a genetic diagnosis of BHD syndrome in a family with a history of spontaneous pneumothorax in 24% of the patients and bullosa in 54% of the families.^[10]^ The discovery of the FLCN gene is a big step forward in the study of the genetic mechanisms underlying primary spontaneous pneumothorax.

Abolnik et al retrospectively analyzed 15 families and proposed that the disease is inherited in an autosomal dominant manner,^[11]^ which has been confirmed by subsequent studies. In 2002, Nickerson et al first reported susceptible pathogenic genes for BHD syndrome, namely FLCN gene.^[5]^ A large number of molecular biological experiments have found that there are 14 exons in the FLCN gene, and the mutation sequence in the patient in this study was located in exon 13.^[6,8,12]^ BHD syndrome is a rare autosomal dominant inherited disorder. It is characterized by repeated spontaneous pneumothorax, multiple lung cysts, multiple skin fibrocystadenomas, and kidney tumors. BHD syndrome is usually characterized by recurrent episodes of spontaneous pneumothorax caused by multiple pulmonary cysts and bullae. Repeated spontaneous pneumothorax and multiple lung cysts are usually located in the lower lobe and fissure space or even adjacent to the surrounding pulmonary vessels.^[2,13,14]^ Pulmonary cystic lesions were found on chest CT in most BHD syndrome patients, but the pathogenesis of pulmonary cysts in these patients remains unclear^[5]^ (see Table 1).

**Table 1:** Clinical characteristics of the case.

Case number	Author	Year	Age	Sex	Pneumothorax	Lung cysts	Renal disease	Skin lesion
1	Kunogi et al	2010	35.1 years old	14 males and 22 females	Yes	Yes	No	N#o
2	Benhammou et al	2011	47.0 years old	–	Yes	Yes	Yes	–
3	Ding et al	2015	48.1 years old	26 males and 14 females	Yes	Yes	No	Yes
4	Rossing et al	2017	28.1 years old	12 males and 11 females	Yes	Yes	No	No
5	Iwabuchi et al	2018	47.0 years old	16 males and 15 females	Yes	Yes	No	Yes
6	Minghui Cai et al	2021	44.0 years old	–	Yes	Yes	No	No
7	Jinrui Miao et al	2024	–	4 males and 5 females	Yes	Yes	Yes	Yes
8	This study		28.0 years old	Female	Yes	Yes	No	No

Currently, both conservative treatment and thoracoscopic resection are commonly used to treat recurrent spontaneous pneumothorax due to BHD syndrome. In the study by Toro et al, 75% of patients with BHD syndrome experienced recurrent pneumothorax,^[5]^ and subsequent thoracoscopic surgery was difficult to achieve once recurrent pneumothorax occurred after thoracoscopic surgery. In recent years, in view of the frequent recurrence of BHD syndrome, some scholars have proposed the technique of pleural covering, that is, using the regenerative oxidized cellulose mesh and fibrin glue to cover the entire pleural surface to prevent recurrence of pneumothorax. Ebana et al reported a case of successful application of the pleural covering technique under thoracoscopy to treat recurrent pneumothorax in BHD syndrome.^[15]^ There was no recurrence of pneumothorax on the affected side during follow-up,^[16]^ but the effect of pleural covering technology on recurrent pneumothorax of BHD syndrome still needs to be confirmed by large sample clinical trials, so its application is relatively limited. Therefore, thoracoscopic surgery is the main treatment for recurrent spontaneous pneumothorax of BHD syndrome in clinical practice. In this case, the patient was followed-up for nearly 2 years after surgery, but there was no recurrence of pneumothorax on the affected side after surgery, and the surgical resection effect was obvious.

This case presents an example of a familial cluster of spontaneous pneumothorax. Through literature review and genetic testing, we identified a clinical syndrome caused by a genetic mutation. Through emergency surgery, the patient fully recovered and there was no recurrence. Although our research has clearly demonstrated a significant correlation between this syndrome and genetic mutations, but we have reviewed a large number of literature, and the mechanism of its occurrence and development has not been fully elucidated. Moreover, due to the patient is unable to provide information on the onset of the disease in family members, we are unable to create a complete genetic map.

## Author contributions

**Conceptualization:** Hu Liao.

**Funding acquisition:** Hu Liao.

**Investigation:** Hu Liao.

**Methodology:** Hu Liao.

**Project administration:** Hu Liao.

**Resources:** Hu Liao, Xu Shen.

**Supervision:** Hu Liao, Xu Shen.

**Writing – original draft:** Hu Liao.

**Writing – review & editing:** Hu Liao, Xu Shen.
